# Clinical pharmacokinetics of antiretroviral drugs in Ugandan patients

**DOI:** 10.7448/IAS.15.6.18079

**Published:** 2012-11-11

**Authors:** M Lamorde

**Affiliations:** Makerere University, Kampala, Uganda; Glasgow Resource Limited Scholar 2008 and HIV Research Trust Scholar

## Abstract

Clinical pharmacokinetic data on antiretroviral drugs are scarce in African HIV-1-positive patients. Most available pharmacokinetic data are derived from ethnically distinct Caucasian research volunteers. This presentation will focus on the clinical pharmacokinetics training and research outputs of an HIV Research Trust scholarship recipient in Uganda. The work highlights the need for post-marketing pharmacokinetic studies in the target populations in order to optimize therapy for patients in resource-limited settings.

